# International good practices on central venous catheters' placement and daily management in adults and on educational interventions addressed to healthcare professionals or awake/outpatients. Results of a scoping review compared with the existent Italian good practices

**DOI:** 10.3389/fmed.2022.943164

**Published:** 2022-10-06

**Authors:** Giovanni Mastrandrea, Rachele Giuliani, Elisabetta A. Graps

**Affiliations:** ^1^Centro Regionale Health Technology Assessment (CReHTA) – Agenzia Regionale Strategica per la Salute e il Sociale (AReSS) Puglia, Bari, Italy; ^2^Istituto di Ricerca e Cura a Carattere Scientifico (IRCCS) Istituto Tumori Giovanni Paolo II – BARI, Bari, Italy

**Keywords:** central venous catheter (CVC), CVC placement, CVC daily management, process standardization, educational interventions, CVC bundles, venous access team

## Abstract

This scoping review aims to check the existing international literature related to the placement and management of central venous catheters (CVCs) in adults and compare them with the Good Practices published by the Italian Society of anesthesiology intensive care (hereafter “SIAARTI Good Practices”) and the protocols written by the Italian Expert group on long-term central venous accesses (hereafter “GAVeCeLT Protocols”) and verify the existence of experiences focused on the daily assessment of the implantation site and on educational interventions on awake patients or caregivers to enhance their empowerment. A systematic search approach has been applied. Our composite research question has been primarily defined by the PICO: only patients over 18 years of age with CVC for any clinical reason except for kidney replacement therapy; placement and management of CVCs with procedures recommended by the recent international guidelines/bundles and specific educational interventions are the interventions to be compared with standard CVC placement and management without any educational interventions. In total, two different types of outcomes have been taken into consideration: catheter-related complications rate (A) and patient/caregiver involvement (B). Eligible articles have been limited to Systematic Review OR Meta-analysis OR Guidelines in Human field, focusing on adults, English language only, from January 2015 to December 2020. Searched Medical Subject Headings (MeSHs) Terms were “venous” AND “catheters,” and the correspondence with the designed PICO framework was then checked directly by the authors. A comprehensive search was conducted by two reviewers on 15 February 2021 in four databases, and 32 full-text articles were finally included and qualitatively assessed. The included articles appear to be in line with the indications provided by the available Italian Good Practices and explain the complexity of this procedure. The need to promote the use of bundles and checklists related to CVC placement and dressing procedures comes to light. These organizational technologies can be implemented following the creation of teams dedicated to venous access that are subjected to continuous training. As regards the impact of educational initiatives, implementing paths of health education and proper hospital discharge preparation for both healthcare workers and families increases safety for the patient with CVC.

## Introduction

Since the beginning of our millennium, central venous catheter (CVC) placement techniques, as well as managing procedures, have undergone a considerable breakthrough. In fact, many international guidelines have been published or updated and numerous bundles have been released to perfect and disseminate good practices among all the involved stakeholders highlighting the essential need to cooperate for the success of a CVC system implantation and usage.

In 2003, Provonost et al. (1), proposed daily goals related to intensive CVC management to be shared among multidisciplinary care teams, patients, their families, and caregivers.

The proposed daily assessment approach would have reduced the length of intensive care unit (ICU) stay also through the prophylaxis of complications such as catheter-related bacteremia and ventilator-associated pneumonia.

Based on this approach, the Institute for Healthcare Improvement (IHI) (2) drew up in 2005, the bundle for the prophylaxis of catheter-related infections including the following interventions:

- Hand hygiene
- Maximal barrier precautions upon insertion
- Chlorhexidine skin antisepsis
- Optimal catheter site selection, with avoidance of the femoral vein for central venous access in adult patients
- Daily review of line necessity with prompt removal of unnecessary lines

Following this campaign, Rhode Island Hospitals reported a 74% decrease in central line-associated bloodstream infections (CLABSIs) from 2006 to 2008 and several hospitals reported an entire year or more without a CLABSI in at least one of their ICUs (3).

David Galpern in 2008 highlighted, about primary and secondary prevention of catheter-related infections, that there were 5 elements to be added or changed from the existing protocol on central line placement (4).

The first element was education. The staff of resident physicians and nurses was educated on bloodstream infection—control practices, which included discussions about proper hand washing, use of full-barrier precautions during the central line insertion, appropriate preparation of the skin with Chlorhexidine, avoiding the femoral site if possible, and early removal of all central lines.

The second element affected supply. A central line cart was created that contained all the equipment needed to comply with evidence-based guidelines for central line insertions.

The third element was personnel. A policy was instituted that required nurses to assist in central line insertion. Previously, central lines were placed by the critical care physicians without assistance, unless requested.

The fourth element was standardization. The nurses implemented a checklist to ensure compliance with the evidence-based guidelines. The final element was ongoing monitoring. As these data were collected, feedback was provided on a real-time basis to the practitioners. In practice, in addition to the need to update the guidelines and standardize protocols, David Galpern has been indicating the establishment of a “CVC placement and management TEAM” that would provide for ongoing monitoring through the collection of data in real-time addition, whether on the one hand, the CVCs are still mainly positioned in the protected hospital environment and are mostly managed by hospital staff, and on the other hand, in the last decade, the increased duration of the CVs life cycle for the quality improvement of the materials has made the CVCs mainly home-based devices. Thus, despite their correct use and daily care being increasingly entrusted to patients/caregivers, a standardized educational offer to promote their engagement is still infrequent.

In the above-mentioned framework, it appears that there is not a comprehensive, recently published, review on the EBM updated routine care and maintenance of adult CVC.

Moreover, no national official guideline has been published so far in Italy, but only expert recommendations and opinions have been published: the SIAARTI Good Practices and GAVeCeLT Protocols. As a result, observing standardized indications and using shared recommendations for site selections (please refer to Table 1: Italian (SIAARTI) indications for CVC placement and recommendation for site selections), different working groups in Italy follow different therapeutic strategies, adopt different approaches, and manage patients in different ways.

**Table 1:** Italian indications for CVC placement and recommendation for site selections.

		**Recommendation for site selection**					
WOCOWA (World Conference on Vascular Access) Classification	CICC (Centrally Inserted Central Catheter)	Internal Jugular Vein can be exploited in large part of its extension in the neck, has some disadvantages due to the infectious risk linked in particular to intensive hospitalization and the possible presence of tracheostomy	Supraclavicular Subclavian Vein is a useful option in case of difficulty in finding exit sites with intact skin (e.g., burns).	Subclavicular subclavian vein has lower infectious risk but is related to pneumothorax, hemothorax and pinch-off from passing the catheter through the costo-clavicular ligament	Infraclavicular axillary vein has exit sites far from areas of possible contamination by tracheo-bronchial secretions and lower infectious risk	Anonymous vein allows for a non-collapsible vein approach and lower exit sites in the neck region	External jugular vein occasionally replaces the internal jugular vein
	PICC (Peripherally Inserted Central Catheter)	Basilic vein (1st choice) is usually distant from the vascular-nervous bundle	Brachial veins (II choice) contained in the vascular-nerve bundle	Cephalic vein (III choice) with 90 ° graft in axillary vein			
	FICC (Femorally Inserted Central Catheter)	Femoral vein is burdened by a high infectious risk. It can be used in specific contexts (temporary dialysis catheter in subjects with BMI <28.4, polytrauma, mediastinal syndrome, emergency conditions, uncoagulated or uncooperative patient).					

Indication for CVC placement: administration of medical solutions for infusion with osmolarity above 900mOsm, pH <5 or pH > 9, and irritating drugs.

Due to the wide multifaceted research question, and the multiple study designs of sources to be included, the authors decided to approach a “scoping review” as the most effective method to examine the subject, synthesize new evidence, and identify the gaps in the literature by applying comprehensive and structured searches of the literature to maximize the capture of relevant information, provide reproducible results, and decrease potential bias (5).

This scoping review, carried out as the very first step of the ongoing “PICC Project” (Patient involvement and Images utilization in Central venous catheters Certification) currently under development in Italy by the Regional HTA Center (CreHTA Puglia) of AReSS Puglia and IRCCS Istituto Tumori Giovanni Paolo II of Bari, aims to check the existing international literature related to the placement and management of CVC in adults, to compare it with the Good Practices published by the Italian association of anesthesiology and intensive care (hereafter “SIAARTI Good Practices”) and protocols written by the Italian Expert group on long-term central venous accesses (hereafter “GAVeCeLT Protocols”) and to verify the existence of experiences including the implantation site daily assessment and educational interventions on both awake patients and caregivers to enhance their empowerment.

## Methods

To fulfill our objectives, this scoping review was designed with a systematic search approach based on the Preferred Reporting Items for Systematic Reviews and Meta-Analysis (PRISMA) Statement and Cochrane Collaboration reporting project (6, 7).

Our composite research question has been primarily defined by the PICO (population, intervention, control group, and outcome) framework: only patients over 18 years of age with CVCs for any clinical reason, except for kidney replacement therapy; placement and management of CVCs with procedures recommended by the recent international guidelines and bundles with high strength of recommendation and specific educational interventions addressed to healthcare professionals and/or patients are the interventions to be compared with standard central venous catheter placement and management without any educational interventions. In total, two different types of outcomes have been taken into consideration: catheter-related complication rate (any complication) (A) and patient/caregiver involvement (the ability of patient/caregiver to manage medication; patient psychological distress) (B) (please refer to Table 2).

**Table 2:** PICO framework.

Population	Patients over 18 years of age with central venous catheters for any clinical reason except for kidney replacement therapy.
Intervention	Placement and management of central venous catheters with procedures recommended by the recent international guidelines and bundles with high strength of recommendation and specific educational interventions addressed to healthcare professionals and/or patients
Comparator	Standard central venous catheter placement and management without any educational interventions addressed to healthcare professionals and/or patients on the most updated placement and/or daily management techniques described in the recently published guidelines and bundles with high strength of recommendation
Outcome	[OUTCOMES A] catheter-related complication rate (any complication) [OUTCOMES B] patient/caregiver involvement (the ability of patient/caregiver to manage medication; patient psychological distress)

In February 2021, with the aim to avoid both “planning” or “measurement” bias, the authors decided to focus only on the last 5 years—Published Secondary Sources: Systematic Reviews or Meta-analysis or Guidelines but, at the same time, to adopt the following wide search strategy that made authors confident not to have excluded any potentially interesting article: (Medical Subject Headings (MeSHs) for “venous” AND “catheters,” limited to Systematic Review OR Meta-analysis OR Guidelines in Human field, focusing on adults data, English language only, published from January 2015 to December 2020).

The correspondence with the designed PICO framework was then checked directly by the authors going through the title/abstract screening first and then throughout the full-text direct reading and selection.

A comprehensive search was conducted on 15 February 2021 in the following four databases: *PubMed, Embase, Web of Science*, and *Scopus*. The initial search strategy was first developed in PubMed by a first researcher (GM) and peer-reviewed by a second research librarian (RG). This search was then translated into the other databases' languages. Specific search strings per each approached database are displayed in Table 3.

**Table 3:** Specific databases' string and results.

**Database**	**Search string**	**Results (n. Tot. items found)**
PubMed	((“veins”[MeSH Terms] OR “veins”[All Fields] OR “venous”[All Fields]) AND (“catheter s”[All Fields] OR “catheters”[MeSH Terms] OR “catheters”[All Fields] OR “catheter”[All Fields])) AND ((guideline[Filter] OR meta-analysis[Filter] OR systematicreview[Filter]) AND (humans[Filter]) AND (english[Filter]) AND (alladult[Filter]) AND (2015:2020[pdat]))	99
Embase	venous AND ('catheter'/exp OR catheter) AND ([cochrane review]/lim OR [systematic review]/lim OR [meta analysis]/lim) AND ([article]/lim OR [review]/lim) AND ([adult]/lim OR [aged]/lim) AND [humans]/lim AND [english]/lim AND [2015-2020]/py	66
Web of science	(TI=(venous AND catheter)) AND LANGUAGE: (English) AND DOCUMENT TYPES: (Review)	107
Scopus	TITLE (venous AND catheter) AND PUBYEAR > 2014 AND PUBYEAR < 2021 AND (LIMIT-TO (DOCTYPE, “review”)) AND (LIMIT-TO (LANGUAGE, “English”))	110
	Tot.	382

A total of 382 articles to be evaluated for eligibility were finally identified from the electronic databases. The two authors (GM and RG) independently performed the databases scanning and title/abstract (Ti/Ab) selection.

Disagreements on the eligibility of studies were solved through discussion and further consultation was not necessary.

After the removal of the 116 duplicates, two review authors (GM and RG) independently screened the 266 remaining titles and abstracts of reports identified by electronic databases.

Based on the inclusion/exclusion criteria for title/abstract, 160 articles were excluded and 106 items have been selected for the full-text assessment. Details on the studies and the reasons for exclusion have been recorded identifying 5 main categories of reasons (Table 4).

**Table 4:** Excluded studies characterization.

**Reason for exclusion on the basis of title/abstract**	**Acronym**	**Number of excluded items**
Non-pertinent titles concerning atrial fibrillation	N.P.af	24
Non-pertinent titles concerning children only (pediatric)	N.P. paed	26
Non-pertinent titles concerning kidney replacement therapy	N.P. kidney	22
Non-pertinent titles concerning various topics (miscellaneous)	N.P. mi	67
Non-pertinent titles concerning thrombosis not catheter-related	N.P. tr	21
TOT	160
Exclusion rate	60.15%

In addition, further 3 articles resulting from a snowballing hand search concerning: guidelines, “gray literature” and dissertations, and simultaneously screened by Ti/Ab, were considered relevant and included among those 106 remaining for the full-text assessment, resulting in 109 items overall to be assessed.

Full copies of all potentially relevant papers have been obtained except for 15 articles that could not be retrieved for a full review, leading to 94 full texts available for the screening.

The two authors (GM and RG) independently screened the full papers, identified relevant studies, and assessed the eligibility of studies for inclusion.

Disagreements on the eligibility of studies were resolved through discussion and when a resolution was not possible, a third author (EAG) was involved.

All irrelevant records were excluded and details of the studies and reasons for exclusion have been recorded.

Of the 94 articles, 6 full texts were excluded because of their inappropriate design (different from Syst Review or Meta- analysis), and 58 Systematic Reviews/Meta-analysis have been excluded as non-pertinent full texts. In total, 30 full texts were resulted as eligible.

During the full-text screening, authors also analyzed and extracted a list of interesting items cited in the 30 selected full texts to be furtherly assessed and included if appropriate. Thus, 15 additional records were extracted from the references list and only two were considered relevant and eligible after the full-text assessment, so they have been included in the final eligible articles pool, resulting in 32 full-text final item candidates for the quality assessment.

**Figure 1:**
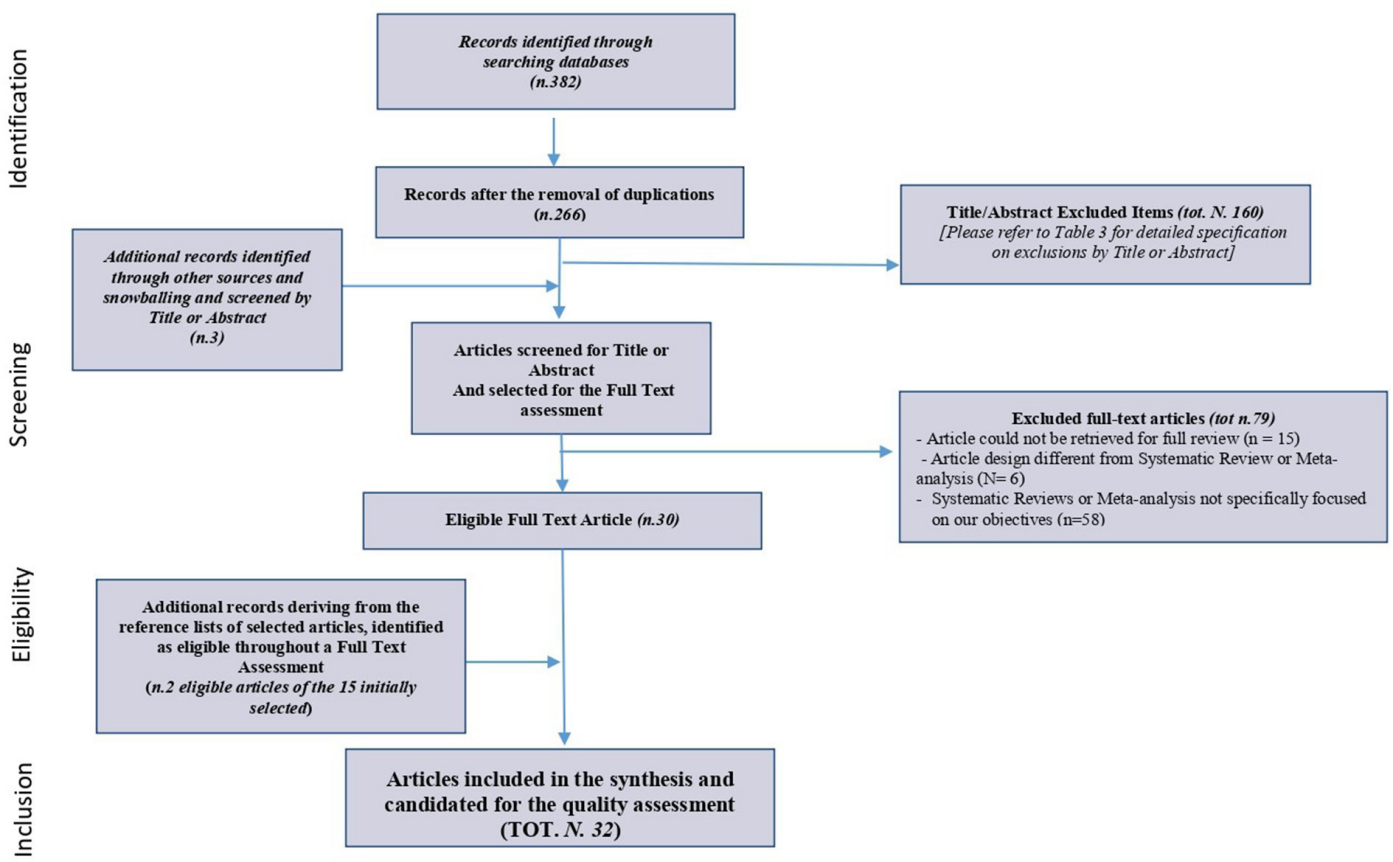
Prisma extension for scoping reviews (Prisma-scr): checklist and explanation.

The PRISMA Extension for Scoping Reviews (PRISMA-ScR): checklist and explanation, presented in Figure 1, describes the whole process.

To fulfill a more effective and tidy results analysis and discussion, eligible articles were split into 5 main thematic categories (Topics) of interest: Topic 1 “*Placement And Imaging*,” Topic 2 “*Dressing*,” Topic 3 “*Infections*,” Topic 4 ”*Educational Intervention Addressed To Nursing And Patients/ Patients Involvement*,” and Topic 5 “*Guidelines On Selection And Care Of Central Venous Access Devices For Adult*.”

According to the pre-assessed inclusion criteria, the selected full texts are secondary sources (Meta-Analysis, Systematic Reviews or Guidelines); however, in the lack of secondary sources available for articles concerning “*Educational Intervention Addressed To Nursing And Patients/ Patients Involvement*” (Topic 4), authors agreed to include and consider the study designs as eligible, different from Meta-Analysis and Syst Reviews. Thus, scoping reviews, qualitative studies (surveys), and prospective cohort studies, which were found out among the screened full text, have been included in the final selection, if eligible.

To summarize, of the selected 32 articles, 20 are Systematic Reviews/Meta-analysis of different topics (from **Topic 1** to Topic 3), 7 are Guidelines (Topic 5), and 5 are different design articles related to Topic 4, educational intervention, and patients' involvement.

Where possible or appropriate, selected articles have been qualitatively assessed using a quality assessment tool properly chosen among those suggested in “Manuale metodologico per la produzione di linee guida di pratica clinica” (8) published by *Centro Nazionale per l'Eccellenza Clinica, la Qualità e la Sicurezza delle Cure CNEC - Istituto Superiore di Sanità (ISS)* on April 2019, according to each specific study design (please refer to Table 5).

**Table 5:** Quality assessment tools.

**Study design**	**Quality assessment tool**	**Score range**
Systematic reviews with/without meta-analysis	AMSTAR II	High Quality: (7 < score <= 16); Low Quality: (0 < = score < 7)
Guidelines	AGREE II	Acceptability: No: Overall Score 1–2 Yes with Modifications: Overall Score 3–5 Yes: Overall Score 6–7
NSR—observational	Newcastle-Ottawa scale	Good Quality: 3 or 4 stars in the Selection domain AND 1 or 2 stars in the Comparability domain AND 2 or 3 stars in the Outcome/Exposure domain Fair Quality 2 stars in the Selection domain AND 1 or 2 stars in the Comparability domain AND 2 or 3 stars in the Outcome/Exposure domain Poor Quality 0 or 1 star in the Selection domain AND 1 or 2 stars in the Comparability domain AND 2 or 3 stars in the Outcome/Exposure domain
Qualitative study	Nice qualitative checklist	++: Score 8.5 to 12 +: Score 4 to 8 –: Score 0.5 to 3.5
Scoping reviews	n.a.	n.a.

The first author developed an extraction table outlining relevant information from the 32 selected articles including Title, First Author, Year of Publication and Journal, as well as the related PICO, when possible, to ensure consistency among reviewers. The second author reviewed and verified the extracted data. A specific extraction table collecting essential references of the 7 Guidelines has been developed separately (Table 6A—Included Studies Extraction Table (other than Guidelines), Table 6B—Included Guidelines Extraction Table).

**Table 6A:** Included studies (other than guidelines) extraction table.

**Progressive Nr**	**Internal code**	**Title**	**First author**	**Journal**	**Year**	**Population**	**Intervention**	**Comparator**	**Outcome**	**Thematic subgroup (topic)**	**Study design**	**Chosen quality assessment checklist**
1	12	Nurses' knowledge on routine care and maintenance of adult vascular access devices: a scoping review	Raynak A	Journal of clinical nursing	2020	Nurses tested in the routine care and maintenance of Adult vascular access devices (VAD)	Practicing nurse seniority (Y/N); work setting (Medicine Units/Surgical Units); gender (M/F); academic background or advanced certification (Y/N); prior workplace training (Y/N)	Practicing nurse seniority (Y/N); work setting (Medicine Units/Surgical Units); gender (M/F); academic background or advanced certification (Y/N); prior workplace training (Y/N)	VAD management knowledge scores	*Topic 4: Educational Intervention Addressed To Nursing And Patients/Patients Involvement*	Scoping review	n.a.
2	29	Prolonging the flush-lock interval of totally implantable venous access ports in patients with cancer: A systematic review and meta-analysis	Wu X	Journal of Vascular Access	2020	862 Oncologic Pts with totally implantable venous access ports (TIVAPS)	Flush-lock interval of TIVAPs > 4 weeks	Flush-lock interval of TIVAPs <= 4 weeks	Total Complication rate, Occlusion (Withdrawal and Total occlusion -withdrawal and flushing), Port-related infections and Mechanical complications	*Topic 2: Dressing*	Syst Rev	AMSTAR II
3	54	Educational practices for families of children and adolescents using a permanent venous catheter	Correa VB	Revista Brasileira de Enfermagem	2020	Children and adolescents	Nurses' educational practice provided to families (home visits, printed educational materials, simulation on mannequins educational videos, combined educational practice)	Standard care without educational intervention	Longterm venous catheters care	*Topic 4: Educational Intervention Addressed To Nursing And Patients/Patients Involvement*	Scoping Review	n.a.
4	90	Comparative efficacy of 13 antimicrobial dressings and different securement devices in reducing catheter-related bloodstream infections: a Bayesian network meta-analysis	Dang FP	Medicine (Baltimore)	2019	8,494 adult Pts	13 Different kinds of antimicrobial dressing and different securement devices for the prevention of CRBSI	13 Different kinds of antimicrobial dressing	CRBSI and CRBSI rate per 1,000 Catheter days, catheter failure	*Topic 2: Dressing*	Syst Rev	AMSTAR II
5	100	8-week interval in flushing and locking port-a-cath in cancer patients: A single-institution experience and systematic review	Fornaro C	Eur J Cancer Care (Engl)	2019	1,347 Pts with port-a-cath (PAC)	Flushing-lock procedure every 4 weeks	Flushing-lock procedure every 8 week	Complications (OCCLUSION, INFECTIONS, AND MECHANICAL DYSFUNCTIONS)	*Topic 2: Dressing*	Syst Rev	AMSTAR II
6	123	Chlorhexidine-impregnated dressing for the prophylaxis of central venous catheter-related complications: a systematic review and meta-analysis	Wei L	BMC INFECTIOUS DISEASES	2019	6,028 Pts	Chlorhexidine-impregnated dressing	Other Dressing/No Dressing	Catheter colonization and CRBSI	*Topic 2: Dressing*	Syst Rev	AMSTAR II
7	175	Review of strategies to reduce central line-associated bloodstream infection (CLABSI) and catheter-associated urinary tract infection (CAUTI) in adult ICUs	Patel PK	J Hosp Med	2018	Non-specified Population—Overall Central-line-associated bloodstream infection—CLABSI-; Catheter-Related Bloodstream Infection - CRBSI- and Central line-associated urinary tract infection CAUTI	Various Interventions on the 4 stages of catheterization (Evaluation−1; Insertion−11, Maintenance-6; Removal-2) OR Interventions to improve Implementation and Sustainability	Various Interventions on the 4 stages of catheterization (Evaluation−1; Insertion−11, Maintenance-6; Removal-2) OR Interventions to improve Implementation and Sustainability	CLABSI per 1,000 catheter days; CRBSIs per 1,000 Catheter Days; Central Line Utilization rates	*Topic 3: Infections*	Syst Rev	AMSTAR II
8	190	Effectiveness of antimicrobial-coated central venous catheters for preventing catheter-related blood-stream infections with the implementation of bundles: a systematic review and network meta-analysis	Wang HL	ANNALS OF INTENSIVE CARE	2018	Non-specified population—overall central-line-associated bloodstream infection—CLABSI-; Catheter-Related Bloodstream Infection—CRBSI-	Antibiotics catheter	Traditional Catheter	CRBSIs per 1,000 Catheter Days; Incidence of catheter colonization	*Topic 3: Infections*	Syst Rev	AMSTAR II
9	192	Bedside ultrasound to detect central venous catheter misplacement and associated iatrogenic complications: a systematic review and meta-analysis	Smit JM	CRITICAL CARE	2018	2,548 pts (2,602 CVC placement) from 25 studies	Bedside Ultrasound	X-ray Chest	Accuracy in detecting CVC misplacement (Specificity and sensitivity of US; the prevalence of malpositioning and pneumothorax, the feasibility of US examination, time to perform and interpret US and X-ray chest)	*Topic 1: Placement And Imaging*	Syst Rev	AMSTAR II
10	199	Assistive technology for ultrasound-guided central venous catheter placement	Ikhsan M	Journal of Medical Ultrasonics	2018	Non-specified Population (any articles on assistive technologies for ultrasound-guided central venous catheterization)	Needle Visualization and guidance (echogenicity); Software enhanced and robot-assisted US-guided CVC placement; ergonomic of US-guided procedures; Alternative and complementary technologies	Commonly used needles; manual ultrasound guided cannulation	Inadvertent penetration of the common carotid artery, repeated penetration of the area, and others	*Topic 1: Placement And Imaging*	Syst Rev	AMSTAR II
11	218	Prevention of central venous line associated bloodstream infections in adult intensive care units: a systematic review	Velasquez Reyes DC	Intensive Crit Care Nurs	2017	Adult patients with CVC in ICU	Chlorhexidine impregnated Dressing, closed infusion systems, Chlorhexidine skin preparation, central venous line bundles (e.g., early removal), quality improvement initiatives, education, extra staff in ICU, participation in the national program for stopping the bloodstream Infections	Other Dressing (honey, standard, sterile gauze, etc.), open infusion systems, H_2_O_2_ or silver skin preparation, etc.	CRBSI rate	*Topic 3: Infections*	Syst Rev	AMSTAR II
12	241	Minimizing central line-associated bloodstream infection rate by inserting central venous catheters in the adult intensive care units	Hina HR	Journal of Clinical Nursing	2017	Adult patients with CVC in ICU	Accurate choice Site of CVC insertion (subclavian), decolonizing patients' skin with cutaneous antiseptic agents (alcoholic chlorhexidine gluconate preparation)	Internal jugular or femoral veins CVC insertion	CLABSI rates	*Topic 3: Infections*	Syst Rev	AMSTAR II
13	259	Knowledge Level on Administration of Chemotherapy through Peripheral and Central Venous Catheter among Oncology Nurses	Kapucu S	Asia-Pacific Journal of Oncology Nursing	2017	Nurses of Turkish Oncology Nursing Society	Questionnaire on sociodemographic aspects and knowledge level on CVC management	n.a.	Correct answers to the knowledge questions	*Topic 4: Educational Intervention Addressed To Nursing And Patients/ Patients Involvement*	Descriptive Study	NICE
14	261	Use of Contrast-Enhanced Ultrasound for Confirmation of Central Venous Catheter Placement: systematic review and meta-analysis: systematic	Bou Chebl R	Journal of Ultrasound in Medicine	2017	Adult patients receiving an internal jugular or subclavian central venous catheter in ICU or Emergency department	Tip location checked with the use of the agitated saline-contrast enhanced US technique	Chest Radiography	Sensitivity and specificity; positive/negative predictive value to confirm the placement of central venous catheters	*Topic 1: Placement And Imaging*	Syst Rev	AMSTAR II
15	278	Diagnostic accuracy of central venous catheter confirmation by bedside ultrasound vs. chest radiography in critically ill patients: a systematic review and meta-analysis	Ablordeppey EA	Critical Care Medicine	2017	Non-specified Population (critically ill patients with CVC)	Bedside US for confirmation of central venous catheter position and exclusion of pneumothorax	Chest Radiography	Accuracy of confirming catheter positioning and detecting a pneumothorax; feasibility, inter-rater reliability, efficiency to complete bedside ultrasound confirmation of central venous catheter position	*Topic 1: Placement And Imaging*	Syst Rev	AMSTAR II
16	287	Environmental exposures and the risk of central venous catheter complications and readmissions in home infusion therapy patients	Keller SC	Infection Control and Hospital Epidemiology	2017	222 Patients discharged with home infusion Therapy	Patients' involvement in self-evaluation (monthly telephone surveys while CVC was in place)	n.a.	30-day readmissions and CVC complications	*Topic 4: Educational Intervention Addressed To Nursing And Patients/ Patients Involvement*	Prospective cohort	New Castle-Ottawa
17	288	Gauze and tape and transparent polyurethane dressings for central venous catheters	Webster J	Cochrane Database Syst Rev	2016	Hospitalized adults and children	Transparent polyurethane dressing	Gauze and tape or other polyurethane dressings	CVC-related infection, catheter security, tolerance to dressing material, and dressing condition	*Topic 2: Dressing*	Syst Rev	AMSTAR II
18	292	Skin antisepsis for reducing central venous catheter-related infections	Lai NM	Cochrane Database Syst Rev	2016	3,446 Pts with CVC (mainly adults admitted to ICU, hematology oncology units, or general wards)	Skin antisepsis as part of CVC care (any agent alone or in combination)	One or more other skin antiseptic agent(s), placebo or no skin antisepsis	Catheter-related BSI or mortality	*Topic 3: Infections*	Syst Rev	AMSTAR II
19	298	Frequency of dressing changes for central venous access devices on catheter-related infections	Gavin NC	Cochrane Database Syst Rev	2016	All patients with CVC in any healthcare setting	Different frequencies of CVAD dressing changes	Different frequencies of CVAD dressing changes	Confirmed catheter-related bloodstream infections (CRDSI), and suspected CRBSI, all-cause mortality, Catheter Site infection, Skin damage, Pain	*Topic 2: Dressing*	Syst Rev	AMSTAR II
20	342	Contributing factors for a late spontaneous peripherally inserted central catheter migration: a case report and review of literature	Beccaria P	J Vasc Access	2015	Adult Patients with peripherally inserted central venous catheters (PICCs)	n.a. (prospective cohort study observing the effectiveness of several images techniques and patients training on PICC management)	n.a. (prospective cohort study observing the effectiveness of several images techniques and patients training on PICC management)	PICC late migration	*Topic 1: Placement And Imaging*	Syst Rev	AMSTAR II
21	344	Ultrasound guidance vs. anatomical landmarks for subclavian or femoral vein catheterization	Brass P	Cochrane Database Syst Rev	2015	2,030 Participants among Adult Patients and Children (2019 procedures) with femoral or subclavian vein catheterization	The two-dimensional US or Doppler ultrasound (USD) guided catheterization (femoral or subclavian vein)	Anatomical landmark-guided puncture (femoral or subclavian vein)	Inadvertent arterial puncture, hematoma formation, total or other complication rates, overall complications, number of attempts until success or first-time success rates or time taken to insert the catheter.	*Topic 1: Placement And Imaging*	Syst Rev	AMSTAR II
22	345	Ultrasound guidance vs. anatomical landmarks for internal jugular vein catheterization	Brass P	Cochrane Database Syst Rev	2015	5108 Participants among Adult Patients and Children internal jugular vein catheterization	The two-dimensional US or Doppler ultrasound (USD) guided catheterization (internal jugular vein puncture)	Anatomical landmark-guided puncture (internal jugular vein)	Inadvertent arterial puncture, hematoma formation, total or other complication rates, overall complications, number of attempts until success or first-time success rates or time taken to insert the catheter.	*Topic 1: Placement And Imaging*	Syst Rev	AMSTAR II
23	365	Dressings and securement devices for central venous catheters (CVC)	Ullman AJ	Cochrane Database of Systematic Reviews	2015	Adult Patients (= >18 y) with CVC (of any kind) in hospital and/or clinical setting	Heparin flush (all heparinized solutions described in the literature)	0,9% Saline flush	Occlusion rates	*Topic 2: Dressing*	Syst Rev	AMSTAR II
24	Ref. 13 Art 90	Using maximal sterile barriers to prevent central venous catheter-related infection: a systematic evidence-based review	Kent KH	AJIC	2004	Non-specified Population with CVC	Maximal sterile barriers during central venous catheter insertion	Less stringent sterile barrier techniques during central venous catheter insertion	Infectious complication rates	*Topic 2: Dressing*	Syst Rev	AMSTAR II
25	Ref. 17 Art 90	Controlling catheter-related bloodstream infections through a multi-center educational program for intensive care units	Musu M	Journal of Hospital Infection	2017	Non-specified Population with CVC five hospitals in the north and center of Italy—the educational intervention was addressed to the healthcare professionals of the ICUs team	Surveillance and Educational programs aimed at healthcare workers to control infections with Interrupted time-series analysis	n.a.	CRBSI rates	*Topic 4: Educational Intervention Addressed To Nursing And Patients/Patients Involvement*	Prospective cohort	New Castle-Ottawa

**Table 6B:** Included guidelines extraction table.

**Progressive Nr**	**Internal code**	**Title**	**1st author**	**Journal**	**Year**	**Population**	**Main topics**	**Thematic subgroup**	**Study design**	**Chosen quality assessment checklist**
26	31	Central venous catheter-related infections in hematology and oncology: 2020 updated guidelines on diagnosis, management, and prevention by the Infectious Diseases Working Party (AGIHO) of the German Society of Hematology and Medical Oncology (DGHO)	Böll B	Annals of Hematology (2020). Date of Publication: 2020	2020	Cancer Pts	Pathogenesis and risk factors; Pathogens; Diagnosis Procedures and Criteria; Prevention (Education, bundles, and surveillance; sterile precautions, skin antisepsis; CVC replacement; CVC site dressing and anti-infective caps, choice of CVC, sutureless devices, the impact of catheterization site, antimicrobial impregnated CVVCs; System and typical antibiotic prophylaxis; antimicrobial lock solutions) Management (catheter removal, antibiotic lock therapy, Systemic antimicrobial treatment)	*Topic 5: Guidelines*	Guidelines	AGREE 2
27	89	Managing and preventing vascular catheter infections: a position paper of the international society for infectious diseases	Lutwick L	Int J Infect Dis	2019	All Patients with VC	Insertion bundle; catheter maintenance bundle; open vs. closed intravenous infusion systems, management of the CLABSI, CLABSIs in pediatrics, limited resource settings,	*Topic 5: Guidelines*	Guidelines	AGREE 2
28	297	Association of anesthetists of Great Britain and Ireland: safe vascular access 2016	Bodenham Chair A	Anesthesia	2016	All patients with VC	Safety of insertion and removal procedures, Prevention, recognition and management of central venous catheter complications, Infection control, Training experience, Consent, and medico-legal aspects, use of *in situ* long-term devices, ultrasound guidance, service provision for vascular access (organizational aspects)	*Topic 5: Guidelines*	Guidelines	AGREE 2
29	102	American society for parenteral and enteral nutrition guidelines for the selection and care of central venous access devices for adult home parenteral nutrition administration	Kovacevich DS	JPEN J Parenter Enteral Nutr	2019	Adult patients (>18 y) receiving Home Parenteral Nutrition	Type and Catheter Materials; Lumen Number And type; Flush Solution for maintenance; method to manage infections and mechanical complications	*Topic 5: Guidelines*	Guidelines	AGREE 2
30	Snowballing 1	ESPEN guidelines on parenteral nutrition: central venous catheters (access, care, diagnosis, and therapy of complications)	Pittiruti M	Clinical Nutrition 28 (2009) 365–377	2009	All Patients with CVC for Parenteral Nutrition	Access, care, diagnosis, and therapy of complications (Appropriateness, how to choose the central venous access device for PN, preferred sites for placement of a central venous access device, the best technique for placement of a central venous access, appropriate position of the tip of central venous access for parenteral nutrition, evidence-based interventions that effectively reduce the risk of catheter-related bloodstream infections, methods for diagnosis of CRBS, and best method for the management of CRBSI in non-tunneled CVCs, the best method for the management of CRBSI in long-term central venous access devices, flushing opportunity, evidence-based recommendations regarding prevention, diagnosis, or treatment of mechanical complications, evidence-based recommendations regarding prevention, diagnosis, or treatment of thrombotic complications.)	*Topic 5: Guidelines*	Guidelines	AGREE 2
31	Snowballing 2	European Society of Anesthesiology guidelines on perioperative use of ultrasound-guided for vascular access (PERSEUS vascular access)	Lamperti M	Eur J Anaesthesiol 2020; 37:344–376	2020	All Patients with VC	Ultrasound-guided cannulation in adults, Ultrasound-guided cannulation in children, ultrasound-guided vascular access for training, Performance indicators for ultrasound-guided vascular access procedures	*Topic 5: Guidelines*	Guidelines	AGREE 2
32	Snowballing 3	Practice guidelines for central venous access a report by the American society of anesthesiologists task force on central venous access	Rupp SM	Anesthesiology, V 116 • No 3	2012	All Patients undergoing elective central venous access procedures performed by anesthesiologists or health care professionals under the direction/supervision of anesthesiologists. The	Placement and management of central venous catheters, reduce infectious, mechanical, thrombotic, and other adverse outcomes associated with central venous catheterization, improve management of arterial trauma or injury arising from central venous catheterization	*Topic 5: Guidelines*	Guidelines	AGREE 2

## Results

In total, 32 full-text final items are the candidates for the quality assessment, detailed information on the quality assessment tools, and the quality score of each selected article, split into the 5 thematic categories (Topics), and are provided in Table 7A (Topic 1: placement and imaging), Table 7B (Topic 2: dressing), Table 7C (Topic 3: infections), Table 7D (Topic 4: educational interventions), and Table 7E (Topic 5: guidelines on selection and care of central venous access devices).

**Table 7A:** Topic 1: Placement and imaging.

**Id Nr**	**Internal code**	**Title**	**First author**	**Journal**	**Year**	**Study design**	**AMSTAR 2 CHECKLIST Score**	**Quality average level**
T1-1	192	Bedside ultrasound to detect central venous catheter misplacement and associated iatrogenic complications: a systematic review and meta-analysis	Smit, JM	Critical Care	2018	Systematic review	10	High
T1-2	199	Assistive technology for ultrasound-guided central venous catheter placement	Ikhsan, M	Journal Of Medical Ultrasonics	2018	Systematic review	1,75	Low
T1-3	261	Use of contrast-enhanced ultrasound for confirmation of central venous catheter placement: systematic review and meta-analysis: systematic	Bou Chebl R	Journal Of Ultrasound In Medicine	2017	Systematic review	3,5	Low
T1-4	278	Diagnostic accuracy of central venous catheter confirmation by bedside ultrasound vs. chest radiography in critically ill patients: a systematic review and meta-analysis	Ablordeppey EA	Critical Care Medicine	2017	Systematic review	7,5	High
T1-5	342	Contributing factors for a late spontaneous peripherally inserted central catheter migration: a case report and review of literature	Beccaria P	J Vasc Access	2015	Systematic review	3	Low
T1-6	344	Ultrasound guidance vs. anatomical landmarks for subclavian or femoral vein catheterization	Brass P	Cochrane Database Syst Rev	2015	Systematic Review	14	High
T1-7	345	Ultrasound guidance vs. anatomical landmarks for internal jugular vein catheterization	Brass P	Cochrane Database Syst Rev	2015	Systematic review	14	High

**Table 7B:** Topic 2: Dressing.

**Id Nr**	**Internal code**	**Title**	**First author**	**Journal**	**Year**	**Study design**	**AMSTAR 2 checklist score**	**Quality average level**
T2-1	90	Comparative efficacy of 13 antimicrobial dressings and different securement devices in reducing catheter-related bloodstream infections: a Bayesian network meta-analysis	Dang FP	Medicine (Baltimore)	2019	Systematic review	9	High
T2-2	123	Chlorhexidine-impregnated dressing for the prophylaxis of central venous catheter-related complications: a systematic review and meta-analysis	Wei L	BMC Infectious Diseases	2019	Systematic review	10,25	High
T2-3	288	Gauze and tape and transparent polyurethane dressings for central venous catheters	Webster J	Cochrane Database Syst Rev	2016	Systematic review	11,25	High
T2-4	298	Frequency of dressing changes for central venous access devices on catheter-related infections	Gavin NC	Cochrane Database Syst Rev	2016	Systematic Review	11,25	High
T2-5	365	Dressings and securement devices for central venous catheters (CVC)	Ullman AJ	Cochrane Database Of Systematic Reviews	2015	Systematic review	13,25	High
T2-6	29	Prolonging the flush-lock interval of totally implantable venous access ports in patients with cancer: a systematic review and meta-analysis	Wu X	Journal Of Vascular Acces	2020	Systematic review	11	High
T2-7	100	8-week interval in flushing and locking port-a-cath in cancer patients: a single-institution experience and systematic review	Fornaro C	Eur J Cancer Care	2019	Systematic review	1,25	Low
T2-8	Ref. 13- art.90	Using maximal sterile barriers to prevent central venous catheter-related infection: a systematic evidence-based review	Kent K	AJIC	2004	Systematic review	4,5	Low

**Table 7C:** Topic 3: Infections.

**Id Nr**	**Internal code**	**Title**	**First author**	**Journal**	**Year**	**Study design**	**AMSTAR 2 checklist score**	**Quality average level**
T3-1	17	Review of Strategies to Reduce Central Line-Associated Bloodstream Infection (CLABSI) and Catheter-Associated Urinary Tract Infection (CAUTI) in Adult ICUs	Patel PK	J Hosp Med	2018	Systematic review	2	Low
T3-2	190	Effectiveness of antimicrobial-coated central venous catheters for preventing catheter-related blood-stream infections with the implementation of bundles: a systematic review and network meta-analysis	Wang HL	Annals Of Intensive Care	2018	Systematic review	10	High
T3-3	218	Prevention of central venous line associated bloodstream infections in adult intensive care units: a systematic review	Velasquez Reyes DC	Intensive Crit Care Nurs	2017	Systematic review	8,5	High
T3-4	241	Minimizing central line-associated bloodstream infection rate by inserting central venous catheters in the adult intensive care units	Hina HR	Journal Of Clinical Nursing	2017	Systematic review	3,5	Low
T3-5	292	Skin antisepsis for reducing central venous catheter-related infections	Lai NM	Cochrane Database Syst Rev	2016	Systematic review	15,25	High

**Table 7D:** Topic 4: Educational intervention addressed to nursing and patients/ patients involvement.

**Id Nr**	**Internal code**	**Title**	**First author**	**Journal**	**Year**	**Study design**	**Checklist score (used checklist)**	**Quality average level**
T4-1	12	Nurses' knowledge on routine care and maintenance of adult vascular access devices: a scoping review	Raynak A	Journal of clinical nursing	2020	Scoping review	–	–
T4-2	54	Educational practices for families of children and adolescents using a permanent venous catheter	Correa VB	Revista Brasileira De Enfermagem	2020	Scoping review	–	–
T4-3	259	Knowledge level on administration of chemotherapy through peripheral and central venous catheter among oncology nurses	Kapucu S	Asia-Pacific Journal Of Oncology Nursing	2017	Descriptive study	10 (NICE check list)	++
T4-4	287	Environmental Exposures and the Risk of Central Venous Catheter Complications and Readmissions in Home Infusion Therapy Patients	Keller SC	Infection Control and Hospital Epidemiology	2017	Prospective Cohort Study	0 (New Castle-Ottawa)	Poor quality
T4-5	Ref. 17 art.90	Controlling catheter-related bloodstream infections through a multi-center educational program for intensive care units	M. Musu	Journal of Hospital Infection	2017	Prospective Cohort Study	3 (New Castle-Ottawa)	GOOD QUALITY

**Table 7E:** Topic 5: Guidelines on selection and care of central venous access devices for adult.

**Id Nr**	**Internal code**	**Title**	**First author**	**Journal**	**Year**	**Study design**	**AGREE II checklist score**	**Quality average level**
T5-1	31	Central venous catheter-related infections in hematology and oncology: 2020 updated guidelines on diagnosis, management, and prevention by the Infectious Diseases Working Party (AGIHO) of the German Society of Hematology and Medical Oncology (DGHO)	Böll B	Annals of Hematology	2020	Guidelines	3	Yes, with implementations
T5-2	89	Managing and preventing vascular catheter infections: A position paper of the international society for infectious diseases	Lutwick L	Int J Infect Dis	2019	Guidelines	2	Yes, it is a Bundles Collection
T5-3	297	Association of Anesthetists of Great Britain and Ireland: Safe vascular access	Bodenham Chair A	Anesthesia	2016	Guidelines	3	Yes, with implementations
T5-4	102	American Society for Parenteral and Enteral Nutrition Guidelines for the Selection and Care of Central Venous Access Devices for Adult Home Parenteral Nutrition Administration	Kovacevich DS	JPEN J Parenter Enteral Nutr	2019	Guidelines	4,5	Yes, with implementations
T5-5	Sb1	ESPEN Guidelines on Parenteral Nutrition: Central Venous Catheters (access, care, diagnosis, and therapy of complications)	Pittiruti M	Clinical Nutrition	2009	Guidelines	3	Yes, with implementations
T5-6	Sb2	European Society of Anesthesiology guidelines on perioperative use of ultrasound-guided for vascular access (PERSEUS vascular access)	Lamperti M	Eur J Anaesthesiol	2020	Guidelines	6	Yes
T5-7	Sb3	Practice Guidelines for Central Venous Access A Report by the American Society of Anesthesiologists Task Force on Central Venous Access	Rupp SM	Anesthesiology	2012	Guidelines	6	Yes

Guidelines have been qualitatively analyzed using AGREE II checklist, and the related results are shown in Table 7E. According to the authors' assessment, the selected guidelines produced scores between 3 (“Acceptable with modifications”) and 6 (“Acceptable”).

The guidelines that received the highest qualitative evaluation (AGREE II score 6) are T5-6, which exclusively focused on the ultrasound guidance, and T5-7 that addressed wider aspects relating to the placement and management of CVCs.

The guideline T5-4 follows with a score of 4.5, the guidelines T5-1, T5-3, and T5-5 have been assessed with the score of 3, and T5-2 follows with a score of 2.

As regards “Placement and Imaging” topic (Topic 1), there are 7 Systematic Reviews/Meta-analysis included and qualitatively assessed through the Amstar 2 Checklist scoring (scoring scale: from 0 to 16), please refer to Table 7A. In total, 7 articles have been split into 2 qualitative categories: high-quality articles (7 < score < = 16) and low-quality articles (0 < = score < 7). There are 4 high-quality (T1-1, T1-4, T1-6, and T1-7) and 3 low-quality articles (T1-2, T1-3, and T1-5).

Topic 2 assesses the dressing phase with 8 included articles. The high-quality level has been assigned to 6 of the 8 articles (T2-1, T2-2, T2-3, T2-4, T2-5, and T2-6), in the light of the Amstar 2 Checklist obtained scores (which may vary from 9 to 13.25); the 2 remaining articles (T2-7 and T2-8) fall into the low-quality level (scoring 1.25 and 4.5 respectively); please refer to Table 7B.

All studies concerning strategies to reduce the incidence of central line bloodstream infections, or to improve infection management, have been gathered together into Topic 3. In total, five systematic reviews have been included, and three of them (T3-2, T3-3, and T3-5) have been assessed through Amstar checklist score as high level of quality with the following scores 10, 8.5, and 15.25, respectively. The articles T3-1 and T3-4 have been assessed as low-quality systematic reviews.

The last topic (Topic 4) aims at assessing the impact of educational interventions addressed to both healthcare professionals and patients/caregivers on CVC daily management; moreover, it also seeks to evaluate the effect of promoting patient involvement. In total, five articles with different study designs have been here included, specifically, two scoping reviews (T4-1 and T4-2), not eligible for any qualitative assessment, because of their specific design; one descriptive study (T4-3) was qualitatively assessed with the NICE checklist and evaluated with a score of ++; two prospective cohort studies (T4-4 and T4-5) were qualitatively assessed using the New Castle Ottawa checklist score and resulted of POOR QUALITY and GOOD QUALITY, respectively. To pursue the research objective, the screened and selected international guidelines (Topic 5) have been analyzed first.

Subsequently, to verify whether any recently published Systematic Reviews and Meta-analysis have highlighted something furtherly relevant; in terms of best practices and recommendations concerning “Placement and Imaging,” “Dressing,” and “Infections,” we have been throughout the results of the Systematic Reviews and Meta-analysis from Topic 1 to Topic 3, including considerations about what has previously come to light from the guidelines. In the end, the results from educational interventions toward the patient and healthcare team, included in Topic 4, have been taken into consideration.

## Guidelines on selection and care of central venous access devices for adult (topic 5)

A recommendation checklist for immediate and clinical practice-oriented usage is provided by Rupp et al. (T5-7). The recommendation list can be divided into 3 different sections, each one describing a specific phase of the placement procedure:

- Section 1: “Before” → 8 recommended actions
- Section 2: “During” → 6 recommended actions
- Section 3: “After” → 6 recommended actions.

The very first action of “before placement phase consists in providing awake/outpatients with the necessary preliminary information about the catheterization, obtaining patient's consent form signature, and accurately filling out the patient's medical chart including all anamnestic information. When procedures are conducted in a multidisciplinary team, to share all information concerning the placement and management of the CVC among professionals and with patients/caregivers is considered of paramount importance to prevent catheter-related complications and injury in general. Particular attention should be paid to the “Allergy assessment” (especially to Latex, Lidocaine, or Heparin) taking into account, if necessary, the possibility of carrying out the implantation procedure in the operating room.

Hand hygiene is the second milestone, recommended in all the guidelines. Hand hygiene is mandatory for both the operator and the assistant, meaning that any healthcare professional attending the CVC-related procedures should take care to carry out accurate hand hygiene. Pittiruti et al. (T 5-5) consider hand hygiene one of the most evidence-based and cost-effective maneuvers for reducing the risk of catheter-related infections.

The third issue is the optimal choice of the insertion/implantation site. The choice of the CVC implantation site should be driven by (a) the patient's therapeutic path (type of therapy and duration), (b) the patient's characteristics (vascular anatomy, comorbidities, daily activities, quality of life, personal requests), and (c) the type of catheter to be implanted (peripheral, central or femoral insertion, tunneled or non-tunneled, and totally implanted or not). In this regard, Lamperti et al. (T5-6) recommend pre-procedural ultrasound evaluation of the vessel and recognition of possible local disease. In a well-organized therapeutic path, an ultrasound vascular assessment could also be included in a pre-hospitalization assessment to guide the choice of the device to be implanted and facilitate the catheter's placement before the start of therapy. It is essential to evaluate the vascular anatomy of the patient (especially the diameter of the vein in which to place the catheter) and the absence of lesions (especially thrombotic) affecting those vascular districts in which the catheter's placement may lead to injuries or worsening of an existing disease.

Once the optimal insertion site has been chosen and an ultrasound vascular assessment has been carried out, the following and fourth essential action is the “skin preparation.”

Skin preparation should be performed using chlorhexidine in alcoholic solution or through povidone-iodine in alcoholic solution, or, as stated by Böll et al. (T5-1), using octenidine/propranolol solutions. The difference between chlorhexidine in alcoholic solution and povidone-iodine in alcoholic solution is related to the different times of action: the chlorhexidine solution (to which some patients show sensitivity) has a faster mechanism of action and persistent activity despite exposure to bodily fluids than the povidone-iodine solution in ensuring adequate antisepsis (9). The use of colored antiseptic solutions helps in the complete antisepsis of the area where the CVC is to be implanted.

Maximum sterile barriers are foreseen in all guidelines and placement protocols of the CVC. In particular, Rupp et al. specify that it is necessary to use an operator-wearing hat (the sterility of the hat in other guidelines does not appear to be necessary), mask (the sterility of the mask does not appear to be necessary for other guidelines), sterile gloves, and sterile gown, while all the guidelines and protocols agree on covering the patient's body with a sterile drape. The use of real-time ultrasound guidance during placement makes it necessary to include the probe cover for the ultrasound probe inside the maximum sterile barriers. In the end, procedures that close the preoperative phase and start the CVC placement intervention are confirmations of the patient's personal data and of the procedure to be performed, the marking of the insertion site, the correct patient positioning, the assembly of equipment/supplies including method of evaluating the position of the tip, and the check of the labeling on all medication and syringes.

About “during” placement phase, all hospitals should have specific standard operating procedures (SOPs) for insertion and removal of vascular access devices including how to draw up clear documentation from insertion to removal. Finally, it is considered that clinicians should review processes to improve the safety and proficiency in vascular access and initiate regular audits to assess compliance with the standards identified in this and other guidance. In short, the difficulties in standardizing implantation procedures seem to be underlined by all the references in the literature.

A 6-points step-wise approach is recommended: pre-procedural ultrasound evaluation of the vessel and recognition of possible local disease; ultrasound-guided real-time puncture; verification of the direction of guidewires and catheters into the vessel chosen; verification of the correct position of the catheter tip; detection of possible post-procedural early and late complications.

The use of real-time ultrasound guidance during the placement procedure should be routinely used and is recommended by most guidelines. There is compelling evidence that ultrasound-guided venipuncture (by real-time ultrasonography) is associated with a lower incidence of complications and a higher rate of success than “blind” venepuncture.

In regard to the verification of the correct position of the catheter tip, assessment techniques include post-insertion chest X-ray, real-time fluoroscopy (which remains the gold standard for imaging), and both intracavitary EKG and electromagnetic guidance. Ultrasound (US) can confirm catheter position with both the real-time supraclavicular technique (SCU) and the transthoracic (TTE) and transoesophageal echocardiographic (TEE) views. Fluoroscopy, while exposing the patient to radiation, allows to perform intraoperatively the position and navigation of the tip and provides a complete image of the entire course of the catheter.

Pre-usage control of the catheter using ultrasound and radiological techniques can be usefully added to intraoperative placement assessment techniques. The final check mainly concerns the careful elimination of devices that were used for CVC placement (guide wire, peel-away introducer, etc.), the verification of the catheter flow and closure (functioning of needle-free connectors), and the fixation system (which clamps the CVC without resorting to stitches, preventing infections).

The “after” phase corresponds to the “life” of the device, and it starts with the first dressing and ends when the catheter is removed. It essentially includes dressing changes that can be carried out even one time a week by applying the date on the dressing or producing a report specifying the status of the exit site and the methods used. A sterile transparent and semipermeable dressing is preferred over sterile gauze that can be better used if the patient is diaphoretic or the site is actively bleeding or oozing. The maintenance of asepsis during all CVC approach maneuvers is to be considered one of the strongest and most supported recommendations in the literature. To ensure the sterile technique during catheter uses, catheter hubs, connectors, and injection ports should be disinfected with alcoholic chlorhexidine, 70% alcohol solution, or an iodophor while applying mechanical friction before access.

Catheter wash must be carried out each time the catheter is used and the evaluation of the state of the device and the patient's condition should be done daily. This period is also affected by the healthcare organization and the existing network between the hospital and the patient's home. Confirmation of proper functioning of the CVC should be indicated at the end of each catheter handling procedure and be verified before each use.

It is therefore suggested that the same “venous access team” (VAT) that placed the catheter should take care of its dressing and its controls. The VAT should, for example, avoid unnecessary catheterization and remove CVCs no longer required, implement education programs and bundles for nurses and physicians including continuous surveillance and feedback, and check the need for the line to be reviewed daily (Böll et al., T5-1). These evaluations, to be constant and effective over time, should be shared with the patient, his/her caregiver, and the health personnel who manage the catheter at the patient's home.

In clinical practice, catheter removal is performed if the device is no longer needed or if the patient does not respond to the treatment of the complication. This is why the guidelines themselves contain many indications relating to the prevention, diagnosis, and treatment of infections. Pittiruti et al. (T5-5) specify that prophylactic administration of systemic or local antibiotics before or during the use of a CVC is not recommended, since it does not reduce the incidence of catheter-related infections. It also states that diagnosis of catheter-related infections is best achieved (a) by the quantitative or semiquantitative culture of the catheter (when the CVC is removed or exchanged over a guide wire), or (b) by paired quantitative blood cultures or paired qualitative blood cultures from a peripheral vein and the catheter, with continuous monitoring of the differential time to positivity (if the catheter is left in place).

Further recommendations concern the staff training, universally recommended as one of the most important and evidence-based strategies for reducing the risk of catheter-related infections [Lutwick et al. (T5-2) and Bodenham Chair et al. (T5-3)].

Particularly, the use of the ultrasound system for the CVC placement requires a specific training course; the ultrasound evaluation must be associated with the ability to insert a needle into a vein, causing as few lesions as possible to the venous wall and maintaining the sterility of the operating field (10). Effective training includes the following: simulation-based teaching (part-task trainers); apprenticeship models; and courses (local/national, basic/advanced).

The Working Party recommends: manikin simulation techniques routinely available to improve novice technique, regular training updates for skill retention, peer tutoring, and online training.

## Placement and imaging (topic 1)

Smit et al. (T1-1), Bou Chebl (T1-3), and Ablordeppey et al. (T1-4) published 3 meta-analyses aimed at synthesizing information regarding the detection of CVC-related complications and misplacement using ultrasound (US) and determining the specificity and sensitivity for confirming the tip location and catheter placement compared to chest radiography.

Smit et al. analysis yielded a pooled specificity of 98.9 (95% confidence interval (CI): 97.8–99.5) and sensitivity of 68.2 (95% CI: 54.4–79.4). US examination was feasible in 96.8% of the cases. The primary outcome was to evaluate the accuracy of bedside US in detecting CVC misplacement.

The prevalence of CVC malposition and pneumothorax was 6.8 and 1.1%, respectively. The mean time for US performance was 2.83 min (95% CI: 2.77–2.89 min), whereas chest x-ray performance took 34.7 min (95% CI: 32.6–36.7 min). Further analyses were performed by defining subgroups based on the different utilized US protocols and intra-atrial and extra-atrial misplacement. US protocols of included studies could be divided into four separate US protocols consisting of (1) vascular US and TTE; (2) TTE combined with contrast-enhanced US (CEUS); (3) a combination of 1 and 2; or (4) supraclavicular US (SCU).

Vascular US combined with TTE was the most accurate.

Bou Chebl et al. showed a pooled sensitivity of 72% (CI 44-91%) and a pooled specificity of 100% (CI 99-100%) and confirmed that in the setting of central venous catheter placement, post-procedural CEUS imaging is a safe, efficient, and highly specific confirmatory test for the catheter tip location compared with chest radiography.

Ablordeppey et al. conclude as well that if the CVC malposition is not detected by ultrasound and concern is high for malposition, such as in the case of multiple cannulation attempts or incomplete/inadequate ultrasound confirmation technique, chest radiography should be performed to rule out catheter malposition.

Beccaria et al. (T1-5) focus on late spontaneous peripherally inserted central catheter migration, confirming the paramount importance of initial malpositioning early identification and the prompt correction. At the same time, the importance to train the patients about device care and management, to avoid behaviors that may compromise the functionality of the catheter, is highlighted.

Brass et al. (in both T1-6 and T1-7) evaluate the effectiveness and safety of two-dimensional ultrasound (US)- or Doppler ultrasound (USD)-guided puncture techniques for subclavian vein, axillary vein and femoral vein puncture, or internal jugular vein, respectively, assessing whether there was a difference in complication rates between traditional landmark-guided and any ultrasound-guided central vein puncture.

All selected articles establish that US-guided placement has become the standard procedure for CVC placement where an ultrasound machine is available, for that it is faster than a chest X-ray and does not expose patients to radiation, despite the current standard of procedure for US-guided CVC placement has a low ergonomics. In addition, needle visibility during the procedure is highly dependent on the practitioner's ability to coordinate the ultrasound probe and the needle in parallel (Ikhsan et al. T1-2).

Ikhsan et al. evaluate, in fact, the existing technological innovation to improve the safety and ease of US-guided central venous catheterization in several fields that are currently the focus of improvements to ultrasound-guided procedures, including needle visualization, software-enhanced, and robot-assisted needle guidance, and improving procedure ergonomics.

Ultrasound-guided CVC placement is possible in the femoral vein; however, a higher priority is given to the results referred to US-guided CVC placement in the subclavian vein and internal jugular vein (IJV), due to a lower potential for catheter-related infections and thrombosis (11).

X-ray has no indications when the catheter tip is located with the intracavitary EKG method if the correct placement is well-recorded by a printed document showing the corresponding P-wave modification; the EKG method itself could be used alone to assess tip position (Beccaria et al. T1-5).

## Dressing (topic 2)

Both the first dressing and the following replacements could be important risk factors for infection. Literature seems to recognize that multiple factors influence the degree of adhesion of the same product to different people's skin. It is also acknowledged that trauma caused by repeated removal and application of adhesives, or adhesive tapes themselves, can cause an erythematous reaction that affects the barrier function of the skin (skin stripping). A compromised barrier function becomes an issue when bacterial overgrowth has been associated with occlusive dressings (such as the less breathable polyurethane dressings), thus preventing skin stripping could be a way to prevent catheter-related infections.

The authors conclude that there are insufficient data to draw a conclusion about whether the frequency of dressing changes influences catheter-related infections, skin damage, pain, quality of life, or cost in patients, and in the absence of clear evidence of an increased risk associated with extending the time between dressing changes, it is reasonable to base decisions on patient preference and costs.

Nevertheless, consistently with what has just been mentioned, Wu et al. (T2-6) clarify that extending flush interval of totally implantable venous access port to longer than 4 weeks is safe and feasible and extending the flush interval to 8 weeks might not increase the incidence of total complications and catheter occlusions.

The same is confirmed by Fornaro et al. (T2-7).

Clinically indicated dressing changes should occur if the dressing is soiled or not intact (Gavin et al. T2-4).

In conclusion, independently from the frequency of dressing, the daily inspection of the exit site remains essential to check for any signs of localized infection.

In the Bayesian network meta-analysis, Dang et al. (T2-1), conclude that transparent dressing may be the best way to prevent catheter-related infections, a sutureless securement device might lead to the lowest incidence of catheter failure specifying that among the commonly used antiseptic agents, chlorhexidine gluconate has been shown to decrease the incidence of catheter-related infections, in addition.

## Infections (topic 3)

In total, two systematic reviews (Patel et al. and Hina et al., respectively, T3-1 to T3-4), highlight the importance of following specific procedural steps to help reduce or prevent catheter-related infections.

Starting from the application of the Institute of Healthcare Improvement reference bundles (providing for: the surgical washing of the hands; the maximum barrier precautions during the placement; the use of chlorhexidine in alcoholic solution for skin antisepsis; the daily evaluation of the CVC; the removal of the device when no more needed and the use of the femoral placement site as a last resort), authors emphasize the importance of antisepsis not only during the CVC placement, but also during the daily management.

According to Lai et al. (T3-5), the use of chlorhexidine in alcohol solution for antisepsis appears to be more effective than iodine povidone in the prophylaxis of colonization and catheter-related infections (reducing the infection rate from 64 cases per 1,000 patients with a CVC with povidone iodine to 41 cases of infection per 1,000 with chlorhexidine).

In addition, Wang et al. (T3-2) state that, with the application of bundles, antimicrobial-impregnated CVCs, chlorhexidine/silver sulfadiazine, silver ions, other antibiotics (5-fluorouracil, vancomycin, benzalkonium chloride, teicoplanin, miconazole/rifampicin, minocycline, and minocycline/rifampin), catheters are more effective than standard non-impregnated CVCs in decreasing the rate of catheter-related infections per 1,000 catheter days and catheter colonization. Chlorhexidine/silver sulfadiazine-coated catheters reduced the rates of catheter-related infections by ~40% and are therefore appropriate for use in patients at high risk of developing catheter-related infections.

Compared to silver ion-impregnated CVCs, chlorhexidine/silver sulfadiazine antiseptic catheters reduce microbial colonization but do not reduce catheter-related infections, although whether or not other antibiotic catheters are superior to chlorhexidine/silver sulfadiazine-impregnated catheters could not be determined.

## Educational intervention addressed to nursing or patients and patients involvement (topic 4)

Raynak et al. (T4-1) specifically focus on evaluating the nurses' knowledge about the routine care and maintenance of adult Vascular Access Devices (VADs) and highlight that there seems to be room for improvement in the educational preparation of nurses and a need for workplace training. Thus, the need to focus on education, on-the-job training, or continuing education in the knowledge of venous access, to improve the quality and the safety of the care, is a priority. The authors, in addition, highlight that identifying the most useful educational strategies is essential and urgent.

The same topic is addressed by Kapucu et al. (T4-3) in a descriptive study. The abovementioned study has been qualitatively assessed using the NICE checklist and obtained the maximum scoring attribution of double plus (++) with the numeric score of 10 (please refer to Table 5). Results from data derived from 165 nurses show that supporting cancer nurses with relevant on-the-job training programs and courses to improve the level of knowledge related to catheter care is extremely important (T4-3).

The majority of nurses who participated in the study answered incorrectly the questions on the frequency of changing infusion sets, or the clothing on the catheter and antiseptic hand sanitation procedures.

Correa et al. (T4-2), in a scoping review, analyzed, on the other hand, the existing scientific literature on educational practices performed by nurses with the families of children and adolescents using long-term venous catheters, specifically concerning home care. Interventions consisted of home visits, production of printed educational materials, use of mannequins for simulation, creation of educational videos, and combined educational practices.

Educational practices are an important alternative to guarantee the autonomy and independence of the individual, aiming at the instrumentalization of individuals or groups in search of improving health conditions. However, it is not enough to follow recommended norms but also and above all, to carry out health education in a process that stimulates inquiry, dialogue, reflection, and shared action. Thus, professionals must know the reality, the worldview, and the expectations of each subject, so that they can prioritize their needs, not just the therapeutic requirements.

The results of this review demonstrate how health education and proper hospital discharge preparation for families of children and adolescents using long-term venous catheters minimize damage and hospitalization due to catheter complications; they also highlight the importance of the nurse's role in this context.

Keller et al., T4-4 article, have been evaluated by the New Castle Ottawa checklist score and assessed as a poor-quality study; it includes 222 patients undergoing chart abstraction and monthly telephone surveys while CVC was in place. It is observed that home infusion therapy is a safe way to receive treatment and is increasingly used to avoid hospitalizations and control costs. Traditional risk factors would play a greater role in complications than home environment risk factors. The authors conclude by saying that more studies related to home infusion therapy would be needed to understand the frequency of complications and how to reduce them.

The last prospective cohort study, Musu et al. (T4-5) has been assessed as a high-quality study. It suggests that implementing educational programs to reduce infection risk is a strategy that works well in a short-time window and could work better if supported by an improved involvement and participation by all staff. They emphasize the need for continuous audit and education *via* constant feedback to all healthcare workers; this would promote long-term adherence to the guidelines and become a consolidated practice among all critical care workers.

## Discussion

A “Comparison Chart,” listing and comparing all key recommendations highlighted in the studies included in our scoping review and those published by the Italian society SIAARTI and GaVeCeLT Expert Group, is provided in Table 8.

**Table 8:** International guidelines, SIAART good practices and GAVeCeLT protcols comparison chart.

**Recommendation from included guidelines and syst reviews**	**SIAARTI good practices reference**	**GAVeCeLT protocols reference**
Pre-, Intra- or Post-Placement US assessment US-guided real-time puncture	Yes	Yes
During placement Maximum sterile barriers –*hand hygiene*, –*sterile gloves*, –*sterile gown*, –*sterile drape*, –*chlorhexidine in alcoholic solution*, –*sterile probe cover for US probe*	Yes	Yes
During placement Tip catheter location –*Intracavitary EKG AND/OR* –*US transthoracic echocardiographic (TTE)* –*US transoesophageal echocardiographic (TEE)*– *contrast-enhanced ultrasonography (CEUS)* Entire catheter location –*real-time fluoroscopy*	Yes	Yes *(real-time fluoroscopy only in difficult placement)*
Post-placement Tip catheter location –*Intracavitary EKG* –*AND/OR* –*US transthoracic echocardiographic (TTE)* –*US transoesophageal echocardiographic (TEE)* –*contrast-enhanced ultrasonography (CEUS)* Entire catheter location –*chest X-ray*	Yes	Yes *(chest X-ray only if necessary)*
Medication *Trasparent semi permeable dressing*	Yes	Yes *(high MVTR—moisture vapor transfer rate)*
Dressing *Sutureless securement device*	Yes *(engineered stabilization device)*	Yes *(subcutaneous anchoring systems in pts with high risk of dislocation)*
Dressing *Needle-free connectors*	Yes *(valved catheter closure systems equipped with Luer-lock coupling)*	Yes
Dressing *Sterile technique during catheter uses*	Yes *(disinfection of the access doors by manual scrubbing with an alcoholic solution for at least 15” OR use port protectors)*	Yes

The large number of studies concerning venous access explains the complexity of this type of procedure, especially because of the frailty of the patients for whom it is practiced and the increasingly longer use of the devices. This complexity is also related to the number of medical nursing teams involved in the placement and management of CVCs. Despite acting in compliance with protocols and guidelines validated at both a national and international levels, the extreme variability of training courses and indications can worsen the difficulties of managing CVCs and further reduce the quality of life of patients with cancer.

Regarding the vascular access/imaging, SIAARTI Good Practices specifies in the Bundle “IMPIANTO” the following important steps:

- hand hygiene before each catheter approach (placement, management);
- maximum system barrier precautions;
- skin disinfection with 2% chlorhexidine-based alcoholic solutions;
- optimal venipuncture site (avoid femoral vein or peripherals veins of the lower limbs where possible);
- ultrasound-guided system. The use of the ultrasound during the placement phases is indisputable: (a) it allows for evaluating the patient's vascular anatomy and choosing the implantation vein and the caliber of the device, carrying out, so, a first thrombosis prevention maneuver; (b) it allows to minimize the number of vein punctures, carrying out; moreover, a second thrombosis prevention maneuver; to reduce damage to the vessels wall, with a consequent reduction of bleeding, also prevents possible exit-site infections; (c) it allows to evaluate the position of the wire, the course of the catheter (if the thoracic areas occupied by the lungs are excluded) and the position of the tip through echocardiographic techniques, where specific manual skills are required.

Speaking about the tip location methods, they can be classified into intraprocedural (intracavitary electrocardiography, fluoroscopy, echocardiography) or post-procedural (chest X-ray). About their usage, indications provided by the SIAARTI Good Practices correspond to the indications provided by the GAVeCeLT protocols, even if the GaVeCeLT protocols express a total preference for intraprocedural methods and consider radiological methods to be less effective in favor of intracavitary EKG and ultrasound methods.

From an organizational point of view, regardless of the method used, it is essential that in the medical record, it is reported the documentation of the performed controls (copy of chest X-ray or fluoroscopy, print of intracavitary EKG trace or echocardiographic image print).

From our analysis, it emerges that developing a system accountable for the procedure performed during the installation, which would allow all stakeholders to efficiently participate in the same procedures, seems to be necessary.

In this context, “stakeholders” are the followings: the whole clinical team, including the physician who carries out the placement, who performs the dressing, the nurses who use the catheter, the oncologist, the caregiver, and the patient himself.

Regarding the dressing procedures, SIAARTI Good Practices and GAVeCeLT protocols agree on the appropriateness to use aseptic techniques for accessing and/or replacing “needless” connectors.

- aseptic techniques for dressing replacement and any catheter maintenance act;
- catheter washing with 20 ml of sterile physiological solution with pulsating technique at each use;
- daily re-evaluation of the need to maintain the catheter.

From our literature analysis, with regard to the dressing technique, it can be inferred that it would be desirable that a well-defined standardized procedure that envisages the use of appropriate devices was set up and that a shared knowledge among all stakeholders, including patients and caregivers, was built on. The methods for implementing this procedure should be shared by all stakeholders during the entire life of the central venous catheter device.

It is therefore believed that it is necessary to draw up a checklist relating to the dressing procedure that enables the patient and his caregiver to daily assess the catheter's dressing status and possibly communicate any possible alteration found to the VAT of reference.

All authors agree with the need to promote the use of bundles and checklists related to CVC placement and dressing procedures. These organizational technologies can be implemented following the creation of teams dedicated to venous access (VA Team) to be subjected to continuous training.

Speaking of the VA Team and the purpose that the same VA Team may look for the same patient throughout the entire life of his/her CVC, as recommended by international guidelines, can be of great benefit for patients; nevertheless, the above-described organizational model can be effectively implemented for the CVC maintenance of outpatients (e.g., oncologic patients) rather than for very complex and hospitalized patients in ICU. Hospitalized patients, in fact, especially those who stay in the hospital for weeks, would be necessarily assisted by different professionals according to their daily availability in the hospital. We suggest that, in those cases, a specific Standard Operating Procedure must be adopted to optimize efficient sign out and handover methods, in accordance with various therapeutic needs of different patients.

The promotion of the culture of asepsis during the procedures, for example, shared and implemented by VAT professionals and all the stakeholders involved in the placement and medication paths is a guarantee of increasing safety for the patient and his device.

As regards the impact of educational initiatives, all the considered articles converge on the idea that implementing paths of health education and proper hospital discharge preparation for both healthcare workers (in hospital and at home) and families increases safety for the patient with CVC.

## Conclusion

The international guidelines and systematic reviews included in our scoping review appear to be in line with the indications provided by both Italian societies.

The procedural standardization of the CVC placement and its daily management request a strong effort in terms of involvement and training of both professionals and patients/caregivers. This approach seems to be crucial in the attempt to bridge the organizational gap between hospital and home care. A greater attitude to accountability and proper tools need to be implemented in the daily practice and adequately shared among professionals: an intraoperative checklist, certified images from ultrasound techniques, implant ongoing monitoring (e.g., photographically documented daily management and review). Patients' direct involvement can represent the milestone to better manage traditional risk factors that play a great role in complications onset during the home management of the CVCs. Moreover, to overcome and abandon heterogeneous medical practices, to prevent untimely CVC removal or improper re-hospitalizations, is mandatory to foster professionals' capacity building on shared evidence-based (EB) procedures reducing the healthcare costs. In this sense, both health education interventions and hospital discharge proper paths, supported by Information and Communication Technologies (ICT), are important goals to pursue in moving toward integrated care ensuring a better patients' quality of life.

By the way, the specific goal of incoming studies should be to carefully assess the patient's ability and availability to interact with professionals and become so empowered to grow to be an active part of the VAT Team, testing prototypical organizational models based on multidisciplinarity.

## Data availability statement

The original contributions presented in the study are included in the article/supplementary material, further inquiries can be directed to the corresponding author/s.

## Author contributions

All authors listed have made a substantial, direct, and intellectual contribution to the work and approved it for publication.

## Conflict of interest

The authors declare that the research was conducted in the absence of any commercial or financial relationships that could be construed as a potential conflict of interest.

## Publisher's note

All claims expressed in this article are solely those of the authors and do not necessarily represent those of their affiliated organizations, or those of the publisher, the editors and the reviewers. Any product that may be evaluated in this article, or claim that may be made by its manufacturer, is not guaranteed or endorsed by the publisher.
